# Abnormal Anionic Porphyrin Sensing Effect for HER2 Gene Related DNA Detection via Impedance Difference between MWCNTs and Single-Stranded DNA or Double-Stranded DNA

**DOI:** 10.3390/molecules23102688

**Published:** 2018-10-18

**Authors:** Jingheng Ning, Long Liu, Xin Luo, Min Wang, Donglin Liu, Rong Hou, Donger Chen, Jianhui Wang

**Affiliations:** School of Chemistry and Biological Engineering, Changsha University of Science & Technology, Changsha 410110, China; liulong_0808@163.com (L.L.); luoxin_gl@126.com (X.L.); wang_min1993@126.com (M.W.); dong993@163.com (D.L.); hourong0406@163.com (R.H.); chendonger9@163.com (D.C.)

**Keywords:** anionic porphyrin, HER2 gene, electrochemical impedance spectroscopy, DNA sensor, bioanalysis

## Abstract

Human epidermal growth factor receptor 2 (HER2) is a key tumor marker for several common and deadly cancers. It is of great importance to develop efficient detection methods for its over-expression. In this work, an electrochemical impedance spectroscopy (EIS) method adjustable by anionic porphyrin for HER2 gene detection has been proposed, based on the impedance difference between multi-walled carbon nanotubes (MWCNTs) and DNA. The interesting finding herein is that with the addition of anionic porphyrin, i.e., meso-tetra(4-sulfophenyl)-porphyrin (TSPP), the impedance value obtained at a glass carbon electrode (GCE) modified with MWCNTs and a single stranded DNA (ssDNA), the probe DNA that might be assembled tightly onto MWCNTs through π-π stacking interaction, gets a slight decrease; however, the impedance value from a GCE modified with MWCNTs and a double stranded DNA (dsDNA), the hybrid of the probe DNA with a target DNA, which might be assembled loosely onto MWCNTs for the screening effect of phosphate backbones in dsDNA, gets an obvious decrease. The reason may be that on the one hand, being rich in negative sulfonate groups, TSPP will try to push DNA far away from CNTs surface due to its strong electrostatic repulsion towards DNA; on the other hand, rich in planar phenyl or pyrrole rings, TSPP will compete with DNA for the surface of CNTs since it can also be assembled onto CNTs through conjugative interactions. In this way, the “loosely assembled” dsDNA will be repelled by this anionic porphyrin and released off CNTs surface much more than the “tightly assembled” ssDNA, leading to a bigger difference in the impedance value between dsDNA and ssDNA. Thus, through the amplification effect of TSPP on the impedance difference, the perfectly matched target DNA could be easily determined by EIS without any label. Under the optimized experimental conditions, this electrochemical sensor shows an excellent linear response to target DNA in a concentration range of 2.0 × 10^−11^–2.0 × 10^−6^ M with a limit of detection (LOD) of 6.34 × 10^−11^ M (S/N = 3). This abnormally sensitive electrochemical sensing performance resulting from anionic porphyrin for DNA sequences specific to HER2 gene will offer considerable promise for tumor diagnosis and treatment.

## 1. Introduction

DNA biosensors are powerful tools for cancer diagnosis and detection [1] and have been extensively developed by different methods including fluorescence [2], luminescence [3], surface plasma resonance spectroscopy [4] and electrochemistry [5]. In recent years, electrochemical DNA sensors have attracted increasing attention for their outstanding advantages such as inexpensive instrumentation, low detection limit and simplicity due to the ease of obtaining an electrical signal [6,7,8,9,10]. Attributed to the amplification effect on electrical signals, nanomaterials are generally utilized to enhance the sensitivity of electrochemical DNA sensors. Among them, with excellent chemical and thermal stability [11], carbon nanotubes (CNTs) exhibit a great potential since they can facilitate electron transportation due to a large surface-to-volume ratio [12,13,14]. Various DNA sensors using CNTs and graphene-like materials as well as nanocomposites as modified materials for electrodes have been widely reported [15,16,17,18]. To further accelerate the signal transduction and enhance the sensitivity, CNTs are generally functionalized by special molecules in the design of most CNTs-based electrochemical sensors [19].

The unique structure of porphyrin (aromatic and planar molecule) allows it to strongly interact with CNTs through π-π conjugation, and porphyrin/CNTs complexes have been widely prepared for various applications in different fields [20,21,22]. It is considered that the flattening of porphyrin molecules can reduce the distance between porphyrin planes and aromatic rings-based nanomaterials (including CNTs) [23], which will cause an efficient acceleration in the electron transfer between porphyrin/nanomaterial complex and the surface of the electrode [24], exhibiting great promise for applications in the electrochemistry field. In fact, porphyrin has been utilized for the development of electrochemical DNA biosensors based on the formation of a nanocomplex with grapheme [25], which bears a remarkable chemical similarity to CNTs [26,27,28,29,30,31]. It can therefore be assumed that porphyrin/CNTs complex may also be a promising candidate for the construction of electrochemical DNA biosensors, which to the best of our knowledge has not been reported so far.

**Scheme 1:**
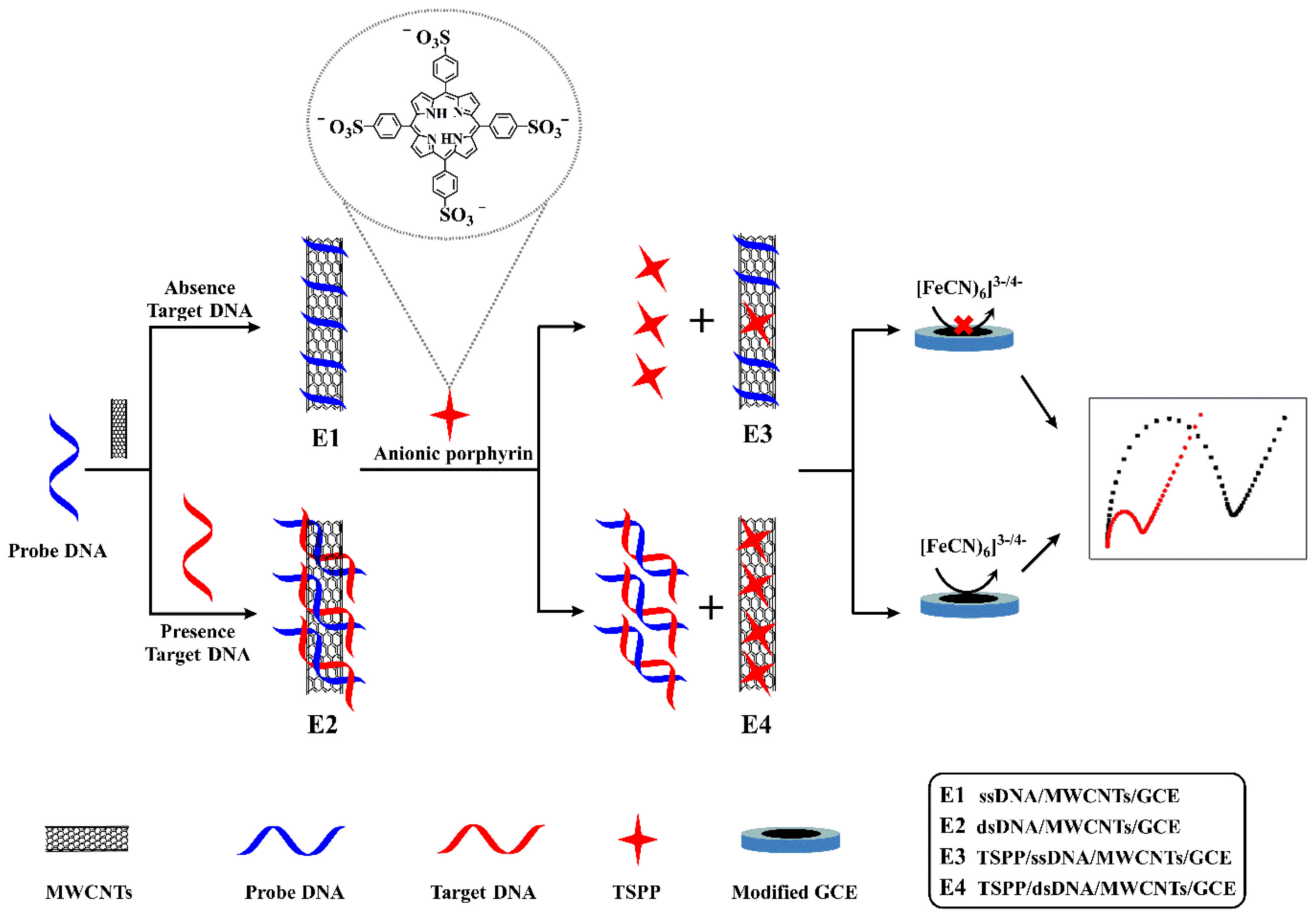
The strategy for DNA detection through electrochemical impedance spectroscopy based on different interactions between anionic porphyrin and different DNA/CNTs complexes.

In view of this, based on MWCNTs and anionic porphyrin, herein we design a simple and sensitive impedance sensor for the determination of DNA fragment specific to the human epidermal growth factor receptor-2 (HER2), which is a tumor marker for the diagnosis of many cancers such as lung cancer, gastric cancer, breast cancer, prostate cancer and ovarian cancer [32,33]. As shown in Scheme 1, firstly, a glass carbon electrode (GCE) is modified with MWCNTs and DNA in the [Fe(CN)_6_]^3−/4−^ redox electrolyte to construct electrochemical platforms, including ssDNA/MWCNTs/GCE (**E1**) and dsDNA/MWCNTs/GCE (**E2**), respectively, where ssDNA (the probe DNA) and dsDNA (a hybrid of the probe DNA and the target DNA) will interact with CNTs differently and probably cause a difference in impedance values. It has been realized that ssDNA can be easily assembled onto the surface of CNTs, owing to the π-stacking interaction between bases in DNA and sidewalls of CNTs. But it is more difficult for dsDNA to be assembled onto CNTs, because of the screening effect of its negatively charged phosphate backbones, which blocks the π-stacking interaction of the bases with CNTs [34,35,36]. Therefore, in this electrochemical platform of **E1**, the probe ssDNA can be tightly assembled onto the surface of MWCNTs and the resulting steric hindrance will block the electron transfer channel of the [Fe(CN)_6_]^3−/4−^, leading to an increase of the impedance value compared with that of the bare GCE. But in the platform of **E2**, after ssDNA has been hybridized by its complementary DNA and turned into dsDNA, the formation of the rigid helix and the screening effect of negative-charged phosphate backbones will weaken the above-mentioned π-stacking interactions and compel dsDNA to be assembled loosely onto MWCNTs, leading to a slight decrease of impedance value compared with that of ssDNA. Thus, an impedance difference between **E1** and **E2** will be obtained, but in such situation is still too small to discriminate ssDNA (the probe DNA) from dsDNA (containing the target DNA).

Then, to enlarge the impedance difference, anionic porphyrin will be utilized due to its “natural” electrostatic repulsion with DNA [37] and its strong conjugative interaction with CNTs [20]. So, as shown in Scheme 1, secondly, an anionic porphyrin, meso-tetra(4-sulfophenyl)-porphyrin (TSPP), is added to the system of **E1** or **E2** to construct an electrochemical platform of TSPP/ssDNA/MWCNTs/GCE (**E3**) orTSPP/dsDNA/MWCNTs/GCE (**E4**), respectively, where TSPP will rival with DNA for the surface of CNTs and cause the decrease of impedance values. The “loosely assembled” dsDNA in **E4** will be repelled by TSPP and released from the surface of CNTs much more than the “tightly assembled” ssDNA in **E3**, leading to a greater reduction of the impedance value as compared with that of ssDNA, and eventually enlarging the impedance difference between **E3** and **E4** to be big enough for the discrimination of ssDNA and dsDNA. This may be caused by the fact that on the one hand, anionic porphyrin will “push” the loosely-assembled dsDNA away from MWCNTs due to the strong electrostatic repulsion between it and DNA; on the other hand, MWCNTs will “pull” anionic porphyrin toward its surface due to strong conjugative interactions between them. Therefore, the loosely-assembled dsDNA on CNTs surface will be easily substituted by TSPP, while the tightly-assembled ssDNA will not. Thus, with the addition of anionic porphyrin—TSPP, based on different interactions between TSPP and different DNA/CNTs (ssDNA/CNTs and dsDNA/CNTs) complexes, the impedance difference between **E3** and **E4** will be amplified enough for the discrimination of ssDNA and dsDNA.

Thus, based on the abnormal regulating and sensing effect of anionic porphyrin on different DNA/CNTs complexes, this assay presented can hereby establish a new efficient DNA electrochemical sensor for HER2 detection without any label, which will have a broad prospect for cancer diagnosis or treatment. Our previous research on interactions between DNA and anionic porphyrin/MWCNTs complex [20] will provide high feasibilities for the proposed strategy described in Scheme 1. At the same time, this current work will surely offer a novel perspective for sensor fabrication via unconventional addition of anionic porphyrin to generate and amplify impedance disparity instead of utilizing optical and chemical sensing amplification, and it also will enrich the research on the unusual composite system containing anionic porphyrin, DNA and CNTs which will combine the advantages of these three kinds of compounds, especially by using the exceptional sensing effect of anionic porphyrin, which will thus will open and pave many potential avenues towards for various applications in electrochemically biomedical fields.

## 2. Experimental Section

### 2.1. Reagents and Materials

Meso-tetra(4-sulfophenyl)-porphyrin (TSPP) was commercially obtained from Sigma-Aldrich (Osaka, Japan). Multi-walled carbon nanotubes (MWCNTs) were purchased from Jcnano Technology Co., Ltd. (Nanjing, China). Hydrochloric acid (HCl, ~37%), Tris(hydroxymethyl)aminomethane (Tris), ethanol (C_2_H_6_O, 99.8%), KCl, K_3_Fe(CN)_6_ and K_4_Fe(CN)_6_·3H_2_O were provided by Sinopharm Chemical Reagent Co., Ltd. (Beijing, China), and were used without further purification. All chemicals were of analytical grade and double distilled water was used throughout this study.

The DNA oligonucleotides were purchased from Sangon Biotech Co., Ltd. (Shanghai, China) and the sequences were listed in Table 1. Tris-HCl solution (pH = 8.00, 50 mM) containing 0.1 M KCl was used as the hybridization buffer.

### 2.2. Apparatus

All electrochemical measurements were performed on a CHI660E electrochemical workstation instrument (Shanghai Chenhua Instrument Co., Ltd., Shanghai, China) using a conventional three-electrode system with a modified glassy carbon electrode (GCE) as the working electrode, a platinum wire as the counter electrode, and an Ag/AgCl electrode as the reference electrode.

### 2.3. Fabrication of DNA Biosensor

The schematic illustration of the fabrication procedure for electrochemical DNA sensor was shown in Scheme 1. The glassy carbon electrode (GCE) was first polished sequentially with 0.3 and 0.05 μm Al_2_O_3_ slurry, followed by ultrasonic cleaning in water, ethanol and then again in water.

To lower the background and improve the utilization efficiency of DNA, the purchased MWCNTs were further purified according to the literature [38]. Briefly, 50 mg MWCNTs were refluxed in 50 mL of 2 M HNO_3_ for 8 h. Then the resulting suspension was centrifuged at 10,000 rpm for 5 min to remove large MWCNTs. The MWCNTs supernatant was rinsed with double-distilled water to reach neutral pH and collected by filtration membrane. The solid MWCNTs were evenly dispersed in the Tris-HCl buffer solution (pH = 8.00, 50 mM) containing 0.1 M KCl by ultrasonic dispersion. The final concentration was about 1.0 g L^−1^.

In order to assemble DNA and modify the bare GCE, firstly, in a 0.05 M Tris-HCl buffer solution (pH 7.40) containing 0.20 M KCl, 5 μL of MWCNTs suspension was mixed with 5 μL of probe ssDNA (20 μM) or with dsDNA, obtained by the hybridization of 5 μL of probe ssDNA (20 μM) with 5 μL of target DNA (2 μM), respectively. Secondly, this mixed solution was dropped onto the surface of pre-treated GCE to afford modified GCE after the solvent evaporated, named as the modified electrode **E1** (ssDNA/MWCNTs/GCE) or **E2** (dsDNA/MWCNTs/GCE). In the same way, the modified electrode **E3** (TSPP/ssDNA/MWCNTs/GCE) or the electrode **E4** (TSPP/dsDNA/MWCNTs/GCE) could also be prepared by an extra addition of 5 μL of TSPP solution. All modified electrodes were stored at 4 °C before use.

### 2.4. Electrochemical Measurements

All DNA sensors were immersed into an electrochemical cell in 0.05 M Tris-HCl buffer solution (pH 7.40) containing 0.005 M [Fe(CN)_6_]^3−/4−^ and 0.20 M KCl. Electrochemical impedance electrochemical impedance spectroscopy (EIS) is a nondestructive technique, which has been widely used in the biopotential sensors [39], electrocatalysis [40,41] and electrochemical sensors [31]. Herein, EIS was employed for the detection of target DNA. EIS measurements were performed at an open-circuit potential with the frequency ranging from 10^5^ Hz to 1 Hz. The amplitude of the applied sinusoidal potential was 5.0 mV, limited to the formal potential of the redox couple [Fe(CN)_6_]^3−/4−^. The electrochemical cell was housed in a specially shielded cage to reduce stray electrical noise during the measurements and all of the measurements were carried out at room temperature. R_et_ is the electron transfer resistance obtained at a modified electrode. The reported result for every electrode in this assay was the mean value of three parallel measurements. An R (C (RW)) equivalent circuit (Nyquist plots) was used to fit the obtained impedance spectra. Data points are the mean values obtained with three independent experiments and error bars are standard deviations for three repetitive experiments.

## 3. Results and Discussion

### 3.1. Construction and Investigation of the Proposed DNA Sensor

**Figure 1:**
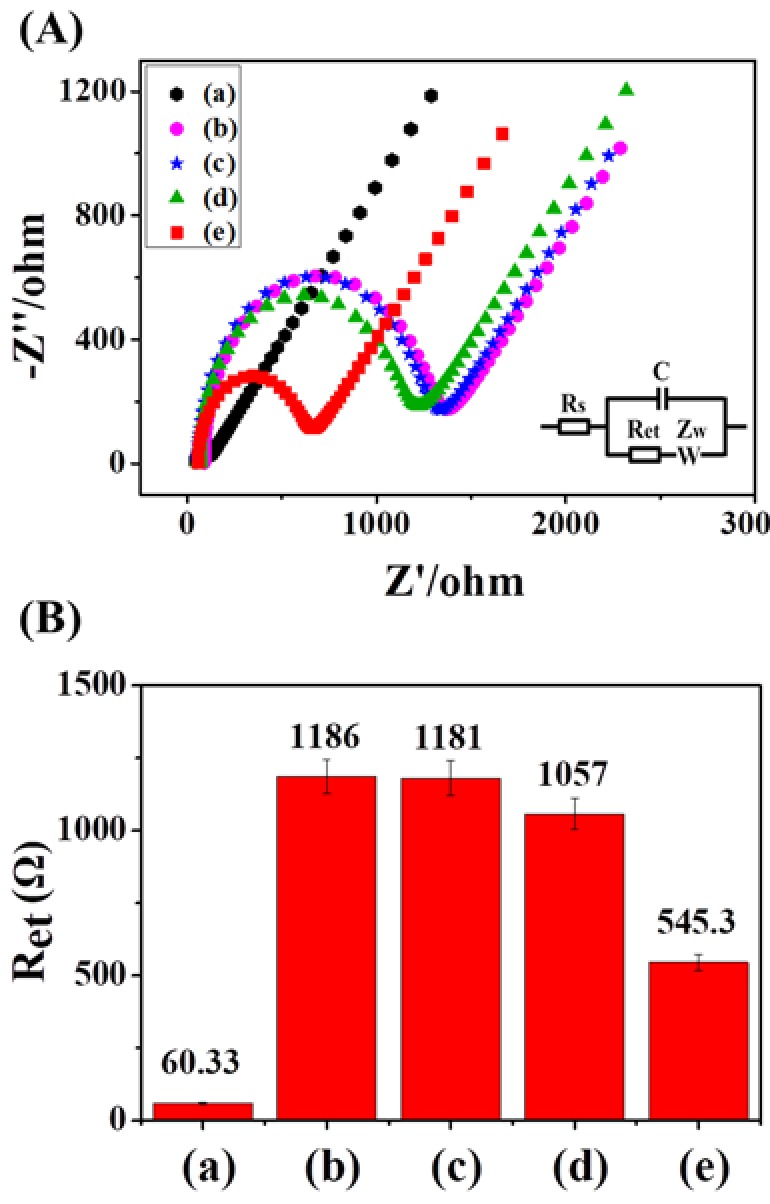
(**A**) Nyquist diagrams from EIS obtained at GCE (a), **E1** (b), **E2** (c), **E3** (d) and **E4** (e) in 0.05 M Tris-HCl buffer solution (pH 7.40), containing 0.005 M [Fe(CN)_6_]^3−/4−^ and 0.20 M KCl. Inset: equivalent circuit used to model impedance data in the presence of redox couples. Rs, C, R_et_ and Z_W_ represent the resistance of the electrolyte solution, the value of capacitance, the interfacial electron-transfer resistance and the Warburg impedance, respectively. The frequency varied from 0.1 to 10^5^ Hz, and 5 mV amplitude of the sine voltage signal was used. (**B**) Histograms for the impedance value of at GCE (a), E1 (b), E2 (c), E3 (d) and E4 (e).

The proposed DNA detection strategy (Scheme 1) was tested for DNA fragments specific to HER2 gene that is a tumor marker related to several kinds of common cancers. Typical electrochemical impedance spectroscopy measurements of the developed technique and the results are shown in Figure 1, and the equivalent circuit component values are listed in Table 2. As defined in Scheme 1, E1, E2, E3 and E4 represent modified electrode ssDNA/MWCNTs/GCE, dsDNA/MWCNTs/GCE, TSPP/ssDNA/MWCNTs/GCE and TSPP/dsDNA/MWCNTs/GCE, respectively.

As can be seen from Figure 1, all of the R_et_ values obtained at modified electrodes **E1** (b, 1186 Ω), **E2** (c, 1181 Ω), **E3** (d, 1057 Ω) and **E4** (e, 545.3 Ω) are larger than that from the bare GCE (a, 60.33 Ω), indicating that based on the interaction with MWCNTs, either DNA or TSPP can be well fabricated onto GCE as designed in Scheme 1. At the same time, R_et,E1_ (1186 Ω) is slightly larger than R_et,E2_ (1181 Ω), indicating that the electron transfer on the electrode/electrolyte interface of **E1** is a little more difficult than that of **E2**. This is because ssDNA can be assembled more tightly onto the surface of CNTs due to strong π-π stacking interactions, so as to block the electron transfer channel of the [Fe(CN)_6_]^3−/4−^ a litter more severely than that in the presence of dsDNA [34,35,36]. And, at this moment, the impedance difference between **E1** and **E2** (ΔR_et_ = R_et,E1_ − R_et,E2_ = 1186 − 1181 = 5 Ω) is very small and provides no chance for target DNA detection.

It is worth noting that in the presence of TSPP, firstly, R_et,E3_ (1057 Ω) and R_et,E4_ (545.3 Ω) decrease in comparison to R_et,E1_ (1186 Ω) and R_et,E2_ (1181 Ω) in the absence of TSPP, respectively, indicating that TSPP can probably substitute DNA and occupy the surface of CNTs through its interaction with CNTs as well as its repulsion of DNA (that is unfavorable for electron transfer) [37]. Secondly, in the presence of TSPP, the impedance difference between **E3** and **E4** (ΔR_et_ = R_et,E3_ − R_et,E4_ = 1057 − 545.3 = 511.7 Ω) is a hundred times bigger than that between **E1** and **E2** (ΔR_et_ = R_et,E1_ − R_et,E2_ = 5 Ω), indicating that TSPP has an excellent amplification effect on the impedance difference and makes it plausible to discriminate ssDNA and dsDNA by EIS. The reason may be that (i) TSPP is rich in planar π-structures like phenyl and pyrrole rings [25], which makes CNTs easily “pull” it onto CNTs surface through strong conjugative interactions; (ii) TSPP is rich of negative charges (four SO_4_^3−^ groups), which makes it naturally “push” DNA far away from it due to strong electrostatic repulsions; (iii) ssDNA can be assembled onto the surface of CNTs more tightly than dsDNA, because there is strong π-stacking interactions between bases in ssDNA and sidewalls of CNTs, but the screening effect of negative-charged phosphate backbones in dsDNA will weaken this kind of π-stacking interactions and loosen the binding of dsDNA with MWCNTs [34,35,36]. Thus, when TSPP is added to the electrochemical platform of DNA/MWCNTs/GCE, TSPP will rival for the surface of CNTs and so the “looser” dsDNA are repelled by this negative porphyrin and released more from the surface of CNTs, leading to a greater decrease of the impedance value (545.3 Ω) compared with that of ssDNA (1057 Ω). Therefore, anionic porphyrin shows an obvious amplification effect on the impedance difference (ΔR_et_) in coordination with CNTs and is a good candidate for the design of electrochemical impedance-discriminative sensors to detect target DNA as described in Scheme 1.

### 3.2. Optimization of Experimental Conditions

**Figure 2:**
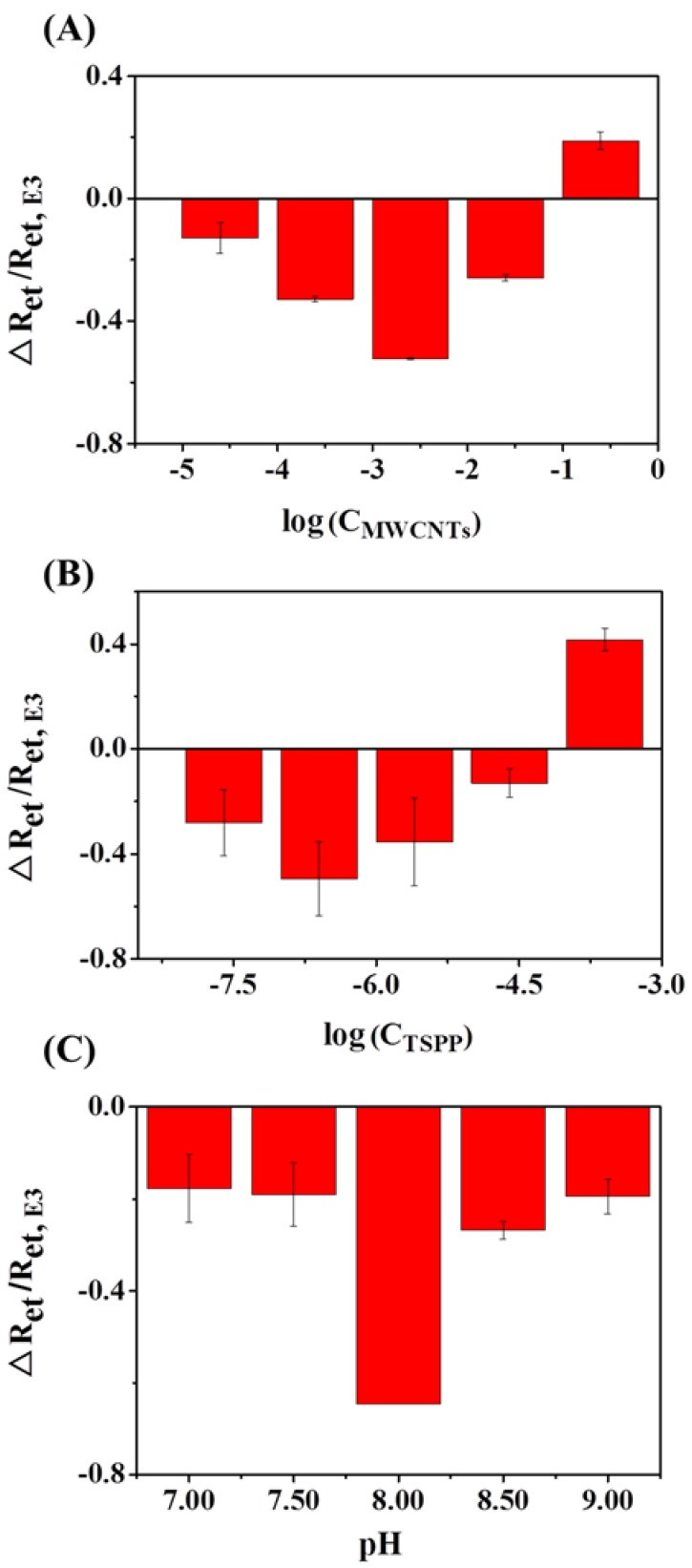
Effect of the concentration of MWCNTs (**A**), TSPP (**B**) solution or the pH value of TSPP solution (**C**) on the relative impedance difference (ΔR_et_/R_et,E3_, herein ΔR_et_ = R_et,E4_ − R_et,E3_) in 0.05 M Tris-HCl buffer solution (pH 7.40), containing 0.005 M [Fe(CN)_6_]^3−/4−^ and 0.20 M KCl. (**A**) The concentrations of MWCNTs solution are 2.5 × 10^−5^, 2.5 × 10^−4^, 2.5 × 10^−3^, 2.5 × 10^−2^ and 2.5 × 10^−1^ g L^−1^; (**B**) The concentrations of TSPP solution are 2.5 × 10^−8^, 2.5 × 10^−7^, 2.5 × 10^−6^, 2.5 × 10^−5^ and 2.5 × 10^−4^ M; (**C**) The pH values of TSPP solution are 7.00, 7.50, 8.00, 8.50 and 9.00.

To further enlarge the impedance difference between **E3** and **E4**, impact factors that affect electrochemical performances of the constructed DNA sensor were sequentially investigated, including the concentration of CNTs or TSPP solution as well as the pH value of TSPP solution (Figure 2). Herein, a ratio of ΔR_et_/R_et,E3_ (ΔR_et_ = R_et,E4_ − R_et,E3_), named as the relative impedance difference, is adopted to provide a more precise representation for the impedance difference between **E3** and **E4**. This is because each impedance value obtained at either **E3** or **E4** was observed to be changing over every different experimental condition; some adjustment must be made in data processing in a way similar to the background correction method. Obviously, the bigger the relative impedance difference (ΔR_et_/R_et,E3_) is, the better the constructed DNA sensor will discriminate the target DNA (existing in dsDNA) from the probe DNA (ssDNA).

Firstly, considering MWCNTs as a necessary substrate for each modified electrode in this work, the effect of the concentration of MWCNTs solution on the relative impedance difference was studied (Figure 2A). As shown in Figure 2A, when the concentration of MWCNTs solution increased from 2.5 × 10^−5^ to 2.5 × 10^−3^ g L^−1^, the absolute value of ΔR_et_/R_et,E3_ gradually increased, due to different interactions between MWCNTs and different DNA. Since neither ssDNA nor dsDNA is favorable for the electron transfer of the [Fe(CN)_6_]^3−/4−^ and the former can interact more easily with CNTs than the latter [34,36], when increasing the concentration of CNTs solution, more and more ssDNA were bound onto the surface of CNTs to cause a greater increase in the impedance value than that caused by dsDNA, producing a bigger and bigger difference in the impedance values between ssDNA and dsDNA. However, after the concentration of CNTs solution was >2.5 × 10^−3^ g L^−1^, the absolute value of ΔR_et_/R_et,E3_ decreased gradually while further increasing the concentration. This is because once the coating of either ssDNA or dsDNA on CNTs surface reaches saturation, the difference in interactions of CNTs with different DNA will be greatly reduced and will not affect the impedance difference as greatly as before. In such a situation, the thickness of the coating materials on the electrode will become a determining impact factor. When further increasing CNTs concentration, both electrodes were overloaded with CNTs and DNA so as to form thick coatings and block the electron transfer channel of the [Fe(CN)_6_]^3−/4−^ to similar extents, thus leading to a smaller and smaller impedance difference. Therefore, results in Figure 2A indicate that the optimal concentration of MWCNTs solution is 2.5 × 10^−3^ g L^−1^, at which the absolute value of the relative impedance difference reaches its maximum.

Secondly, as an important regulatory molecule for the construction of this CNTs-based DNA sensing platform, the effect of the concentration of TSPP on the relative impedance difference was studied (Figure 2B). As can be seen from Figure 2B, when the concentration of TSPP solution increased from 2.5 × 10^−8^ to 2.5 × 10^−7^ M, the absolute value of ΔR_et_/R_et,E3_ gradually increased, mainly caused by different interactions between TSPP and different DNA/CNTs complexes. As described in Scheme 1, MWCNTs can be readily modified by TSPP, which will push DNA away from the surface of CNTs in the meantime [37]. When increasing the concentration of TSPP solution, due to the increasingly strong electrostatic repulsion, more and more loosely-assembled dsDNA on CNTs surface were substituted by TSPP than tightly-assembled ssDNA [20,34,35,36], leading to a greater decrease in the impedance value than that caused by ssDNA, and finally leading to a bigger difference in the impedance values between ssDNA and dsDNA. However, after the concentration of TSPP solution was >2.5 × 10^−7^ M and with its further increase, more and more TSPP molecules were absorbed by CNTs to add the negative charges of the electrode surface [25], which prevented the approach of the negative [Fe(CN)_6_]^3−/4−^ and blocked the electron transfer channel, instead leading to a gradual decrease in the impedance difference. Obviously, the optimal concentration of TSPP solution is 2.5 × 10^−7^ M, which causes a maximum absolute value of the relative impedance difference and is surely most favorable for the discrimination of ssDNA and dsDNA.

Thirdly, under the optimal concentrations of CNTs and TSPP solutions, the effect of the pH value of TSPP solutions on the relative impedance difference was studied (Figure 2C), since the electrostatic and aggregate state of TSPP commonly depends on the medium pH [42], which will influence its electrostatic repulsive interaction with DNA and its loading amount on the surface of MWCNTs. Considering that experiments related to DNA are often carried out under neutral or alkaline conditions, the pH investigated in this work was limited within a range from 7.00 to 9.00, as can be seen in Figure 2C. Results show that from pH 7.00 to 8.00, the absolute value of ΔR_et_/R_et,E3_ gradually increased with increasing pH value of TSPP solution. It is because the deprotonation degree of TSPP will be enhanced with the increase of pH, leading to increases firstly in its electronegativity and then in its repulsive force acting on DNA [20]. Thus, compared with the “tightly-assembled” ssDNA, more and more “loosely-assembled” dsDNA were repelled by more TSPP and released from the surface of CNTs [34,37], leading to a gradual increase in the relative impedance difference. But after pH > 8.00, the absolute value of ΔR_et_/R_et,E3_ decreased with the further increase of pH value, attributed to the fact that the stronger alkalinity will affect the lifetime of the electrochemical sensor. Therefore, as can be seen from Figure 2C, the optimal pH condition for this electrochemical DNA sensor is at 8.00.

### 3.3. Sensitivity of the Proposed DNA Sensors

**Figure 3:**
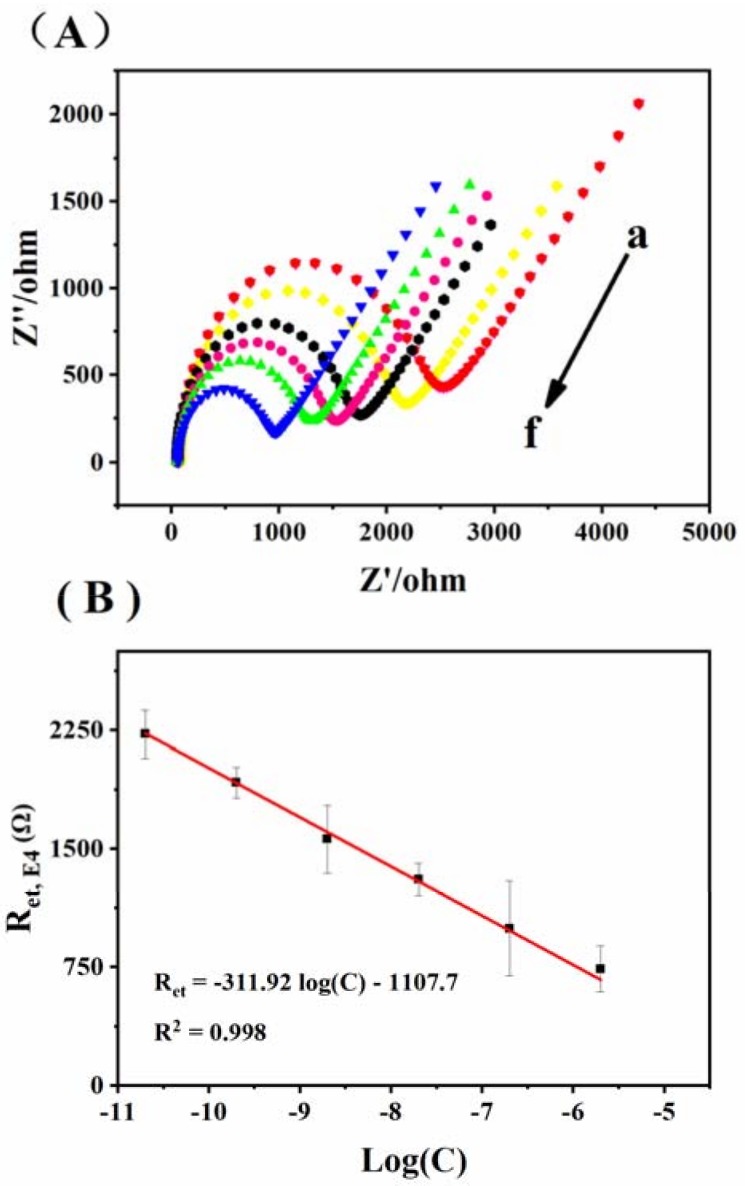
(**A**) Nyquist diagrams recorded at TSPP/dsDNA/MWCNTs/GCE (**E4**) with different concentrations of target DNA: 2.0 × 10^−11^ M (a), 2.0 × 10^−10^ M (b), 2.0 × 10^−9^ M (c), 2.0 × 10^−8^ M (d), 2.0 × 10^−7^ M (e) and 2.0 × 10^−6^ M (f); (**B**) Plot of the relative changes of the impedance value R_et_ versus concentration. Data points are the mean values obtained.

Under the optimized experimental conditions (MWCNTs solution: 2.5 × 10^−3^ g L^−1^; TSPP solution: 2.5 × 10^−7^ M; pH = 8.00), the impedance values of the proposed sensors in different concentrations of target DNA (hybridized with probe DNA to give dsDNA) were investigated (Figure 3). As shown in Figure 3A, R_et_ decreased along with the increase of the concentration of target DNA. The corresponding calibration plot is shown in Figure 3B. It clearly indicates that there is a good linear relationship between R_et_ values and target DNA concentrations across a range from 2.0 × 10^−11^ M to 2.0 × 10^−6^ M with regression equation R_et_ (Ω) = −311.92 log C (M) − 1107.7 and correlation coefficient of 0.998. The limit of detection is found to be 6.34 × 10^−11^ M at a signal-to-noise ratio of 3. These results indicate that the proposed DNA sensor based on MWCNTs and TSPP probably exhibits a high sensitivity for all previously ascribed reasons such as the high specific surface of CNTs, the short distance between porphyrin and CNTs that increases electron transfer [19,23], and especially different attractions or repulsions occurring among CNTs, ssDNA/dsDNA and anionic porphyrin, which produce synergetic effects to amplify the impedance difference to be big enough for target DNA detection without any label. As can be seen from Table 3, compared with some biosensors for the detection of DNA specific to genes from the same family of tyrosine kinase receptor [43,44,45,46,47], such as HER2 (also known as ERBB2 or c-erbB-2) and HER1 (also known as EGFR [48]), our work presents an excellent sensing platform that can be constructed in a quite simple way with good sensitivity and a wide dynamic range (Figure 3).

### 3.4. Selectivity and Reproducibility of the Proposed DNA Sensors

**Figure 4:**
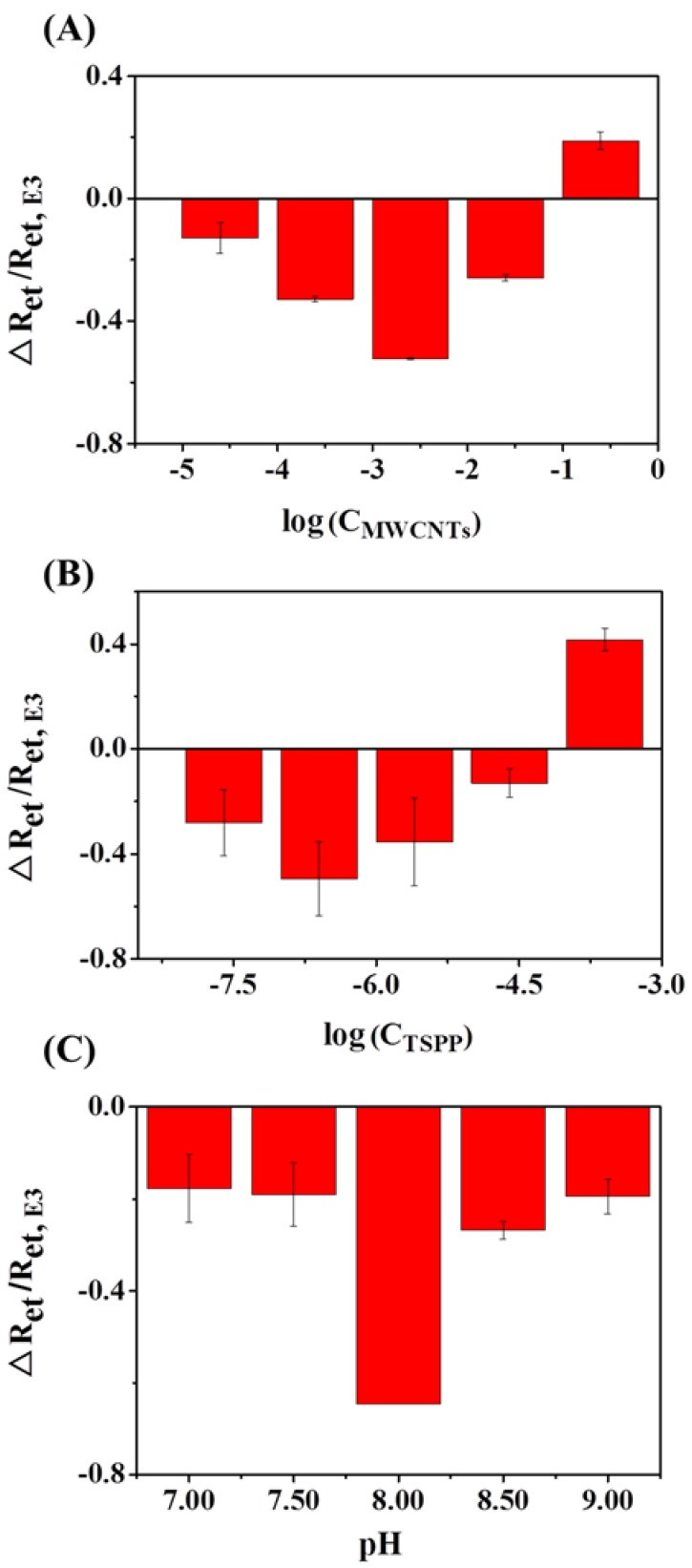
Histogram comparing hybridization signal intensities after detection of 2 μM of perfectly matched target DNA, and one-base mismatched, three-base mismatched and non-complementary target DNA. Measurements were conducted in 0.05 M Tris-HCl buffer solution (pH 7.40) containing 0.005 M [Fe(CN)_6_]^3−/4−^ and 0.20 M KCl.

To demonstrate the capability of our strategy for sequence-selective detection, different DNA sequences were tested at a concentration of 2 μM in 5 mM [Fe(CN)_6_]^3−/4−^, including the perfectly matched target DNA (complementary DNA), one-base mismatched, three-base mismatched, and the non-complementary target DNA (Table 1). The values of ΔR_et_/R_et,E3_ were recorded and shown in Figure 4. Obviously, hybridization is only effective with the nucleotide sequence strictly complementary to the probe DNA. ΔR_et_/R_et,E3_ values obtained from one-base mismatched, three-base mismatched and non-complementary DNA are 43.72, 36.54 and 28.44%, respectively, compared with that obtained from complementary DNA. The results indicate that this impedance electrochemical DNA sensor has reasonable selectivity and can be used to detect DNA sequences related to HER2 gene. In order to characterize the reproducibility of this label-free electrochemical DNA sensor, three trials were run and each modified electrode was freshly prepared. An acceptable relative standard deviation (RSD) of 8.6% (*n* = 3) for ΔR_et_/R_et,E3_ value was estimated, which implies the high reproducibility of the impedimetric DNA sensor.

## 4. Conclusions

In this work, a series of modified electrodes were constructed and studied by means of electrochemical impedance spectroscopy, including ssDNA/MWCNTs/GCE (**E1**), dsDNA/MWCNTs/GCE (**E2**), TSPP/ssDNA/MWCNTs/GCE (**E3**) and TSPP/dsDNA/MWCNTs/GCE (**E4**), for the detection of DNA sequences specific to HER2 gene—a tumor marker related to several kinds of common cancers. Results show that firstly, in the absence of the anionic porphyrin TSPP, the impedance difference between E1 and E2 is too small (5 Ω) to discriminate ssDNA and dsDNA; but secondly, in the presence of TSPP, the impedance difference between E3 and E4 is greatly enhanced (511.7 Ω), demonstrating that TSPP has an outstanding amplification effect on the impedance difference and make it plausible to discriminate dsDNA (containing the target DNA) from ssDNA (the probe DNA).

Then, to further enlarge the impedance difference between **E3** and **E4**, a series of impact factors were investigated including the concentration of CNTs or TSPP solution as well as the pH value of TSPP solution. Results show that the electrochemical platform containing TSPP exhibits an excellent linear response to target DNA in a concentration range of 2.0 × 10^−11^–2.0 × 10^−6^ M with a limit of detection of 6.34 × 10^−11^ M, under optimal experimental conditions with a concentration of MWCNTs solution of 2.5 × 10^−3^ g L^−1^ and a concentration of TSPP solution of 2.5 × 10^−7^ M at pH 8.00.

Therefore, based on the amplification effect on the impedance difference of anionic porphyrin TSPP in the cooperation of CNTs, the perfectly matched target DNA can be easily determined by EIS without any label. This electrochemically efficient label-free DNA sensor will provide a new approach and low cost technique for genetic diagnosis or treatment, and is also significantly essential for the advancement of anionic porphyrin-DNA chemistry. Further studies on the development of DNA biosensors based on impedance difference disparity systems is fundamentally necessary while different nanomaterials modified with various functional molecules are being trialed as expected in our laboratory.

## Figures and Tables

**Table 1:** Oligonucleotides employed in this work.

ssDNA Names	Sequence (5′~3′)
Probe DNA	CCTCACTTGGTTGTGAGCGATGAGCACGT
Complementary DNA(Target DNA)	ACGTGCTCATCGCTCACAACCAAGTGAGG
One-base mismatched DNA	ACGGGCTCATCGCTCACAACCAAGTGAGG
Three-base mismatched DNA	ACGGGCTCATCGCGCAGAACCAAGTGAGG
Non-complementary DNA	CCATTGTCTAGCACGGCCAGGGCATAGTT

**Table 2:** Equivalent circuit component values.

Samples	R_S_/Ω	R_et_/Ω	C/F	Z_W_/Ω^−1^S^0.5^
GCE	43.7	60.33	4.09 × 10^−7^	0.0007519
ssDNA + MWCNTs	88.93	1186	7.55 × 10^−7^	0.0008785
dsDNA + MWCNTs	56.96	1181	7.76 × 10^−7^	0.0009007
ssDNA + MWCNTs + TPPS	63.55	1057	7.96 × 10^−7^	0.0007424
dsDNA + MWCNTs + TPPS	57.93	545.3	8.36 ×10^−7^	0.0008394

**Table 3:** Comparison of some biosensors for the detection of DNA specific to genes that belong to the same family of tyrosine kinase receptor [43].

Analyte	Technique	Material	LOD(M)	Reference
DNA specific to EGFR (HER1) mutations	DPV	HRP modified Ab/DP/tDNA/CP/PPy/AuNP-PWE	1.67 × 10^−10^	[44]
Target DNA of EGFR (HER1)	ECL	AuNPs-probe DNA/target DNA/MPA-CuZnInS QDs-capture DNA PMel/GCE	4.3 × 10^−12^	[45]
DNA species of c-erbB-2 (HER2)	Fluorescence	G/P/T + ThT + Exo III	2.0 × 10^−11^	[46]
Target DNA of ERBB2 (HER2) gene	CA	DNA-c/AuNPs-GO/GCE	1.6 × 10^−10^	[47]
DNA sequences of HER2 gene	EIS	TSPP/DNA/MWCNTs/GCE	6.34 × 10^−11^	This work
